# Cover Picture: Polyamine Ligand-Mediated Self-Assembly of Gold and Silver Nanoparticles into Chainlike Structures in Aqueous Solution: Towards New Nanostructured Chemosensors (ChemistryOpen 5-6/2013)

**DOI:** 10.1002/open.201380501

**Published:** 2013-12-19

**Keywords:** 1D nanochains, self-assembly, gold, mercury, nanoparticles, silver

## Abstract

**Cover Picture:**

Adrián Fernández-Lodeiro, Javier Fernández-Lodeiro, Cristina Núñez*, Rufina Bastida, José Luis Capelo, and Carlos Lodeiro*

**The cover picture shows** the formation of one-dimensional nanochains of silver nanoparticles (AgNPs) upon addition of a polyamine molecular linker. In the present work, the binding ability of this linker was exploited, bearing different functional groups, which favors the self-assembly of silver and gold nanoparticles (AuNPs) into one-dimensional nanochains in aqueous solution. These nanochains visually resemble the artistic work of “calceteiros” (pavers) to build the “calçada Portuguesa” (Portuguese pavements) in Portugal (Photography “calçada Portuguesa”, Jose M. G. Pereira). Polyamine ligands are very versatile materials due to their water solubility and flexibility. The study of AgNPs and AuNPs in one-dimensional nanostructured formations are finally used as new sophisticated heavy metal (Hg^2+^) ion chemosensors. For more details, see the Full Paper by Carlos Lodeiro, Christina Núñez and co-workers, on p. 200 ff.

